# Literary Note

**Published:** 1910-09

**Authors:** 


### LITERARY NOTE

Lea & Febiger, Publishers, Philadelphia, announce a new edition of Gray's Anatomy to be used early in September. Eighteen editions have been demanded in the course of its half century, and they have enlisted many of the ablest anatomists of this period. The principles on which Gray built his book have been followed, and it is not too much to say that during two generations it has guided the teaching of its subject in America as well as England.

Of all the editions, this new one represents the most thorough revision. Every line has been scanned for possible improvement. Anything in the nature of a possible obscurity has been clarified, passages have been rewritten, and new developments have been incorporated. Professor Spitzka, the editor, is one of the foremost anatomists, and he joins to this the apt qualification of being himself an artist as well, so that the drawings from his own hand present his knowledge directly to the mind of the reader. Another of Gray's fundamental improvements, in which his book has always been unique, was the engraving of the names of the parts directly on them, so that the student learned at once not only their nomenclature, but also their position, extent and relations, the four cardinal points. The advantage of this graphic method over the elsewhere customary lines and reference letters is obvious. Gray's book was also the first to contain illustrations in colors. In this new edition, besides all the improvements in the text, the splendid series of characteristic illustrations has been equally revised, many cuts being replaced and more added, and the use of colors is more lavish than ever. No student in any profession, or in any branch of medicine, has offered to him any instrument of instruction comparable to Gray's Anatomy. It suffices to say that the new edition will excel any of its predecessors.

